# Targeting the Main Sources of Reactive Oxygen Species Production: Possible Therapeutic Implications in Chronic Pain

**DOI:** 10.2174/1570159X22999231024140544

**Published:** 2023-10-25

**Authors:** Peng-Fei Cheng, Yuan-He, Meng-Meng Ge, Da-Wei Ye, Jian-Ping Chen, Jin-Xi Wang

**Affiliations:** 1 Division of Colorectal Surgery, Third Hospital of Shanxi Medical University, Shanxi Bethune Hospital, Shanxi Academy of Medical Sciences, Tongji Shanxi Hospital, Taiyuan, 030032, China;; 2 Department of Anesthesiology and Pain Medicine, Tongji Hospital, Tongji Medical College, Huazhong University of Science and Technology, Wuhan, 430030, China;; 3 Cancer Center, Tongji Hospital, Tongji Medical College, Huazhong University of Science and Technology, Wuhan, 430030, China;; 4 Department of Pain Management, Shanxi Bethune Hospital, Shanxi Academy of Medical Sciences, Tongji Shanxi Hospital, Third Hospital of Shanxi Medical University, Taiyuan, 030032, China

**Keywords:** Reactive oxygen species, chronic pain, mitochondria, NADPH oxidase, peroxisome, analgesics

## Abstract

Humans have long been combating chronic pain. In clinical practice, opioids are first-choice analgesics, but long-term use of these drugs can lead to serious adverse reactions. Finding new, safe and effective pain relievers that are useful treatments for chronic pain is an urgent medical need. Based on accumulating evidence from numerous studies, excess reactive oxygen species (ROS) contribute to the development and maintenance of chronic pain. Some antioxidants are potentially beneficial analgesics in the clinic, but ROS-dependent pathways are completely inhibited only by scavenging ROS directly targeting cellular or subcellular sites. Unfortunately, current antioxidant treatments do not achieve this effect. Furthermore, some antioxidants interfere with physiological redox signaling pathways and fail to reverse oxidative damage. Therefore, the key upstream processes and mechanisms of ROS production that lead to chronic pain *in vivo* must be identified to discover potential therapeutic targets related to the pathways that control ROS production *in vivo*. In this review, we summarize the sites and pathways involved in analgesia based on the three main mechanisms by which ROS are generated *in vivo*, discuss the preclinical evidence for the therapeutic potential of targeting these pathways in chronic pain, note the shortcomings of current research and highlight possible future research directions to provide new targets and evidence for the development of clinical analgesics.

## 1 INTRODUCTION

Chronic pain refers to persistent or recurring pain that lasts for an extended period of time, typically exceeding 12 weeks. It affects approximately 20% of the global population and imposes a heavy economic burden amounting to about $635 billion annually [1-3]. Despite significant advancements in designing effective therapeutic pharmacological interventions, chronic pain still poses a significant unresolved healthcare issue [4-9]. Opioids, the mainstay of chronic pain management, have contributed to over 80,000 U.S. overdose deaths in 2021. Thus, new non-opioid approaches are desperately needed [10].

Reactive oxygen species (ROS) and reactive nitrogen species (RNS) are crucial mediators in cell signaling and homeostasis [11]. *In vivo*, one-electron reduction of O_2_ results in the O_2_**^• −^** formation, which subsequently undergoes disproportionation catalyzed by superoxide dismutase to generate O_2_ and H_2_O_2_. The other fate of O_2_**^• −^** is to react with the endogenous production of NO to form ONOO– through a nonenzymatic process [12]. *In vivo*, the generation and scavenging of ROS/RNS are in a state of homeostasis. However, when this balance is disrupted, an excessive accumulation of ROS/RNS occurs, leading to oxidative/nitrosative damage to biomolecules such as lipids, DNA, monosaccharides, and proteins [13, 14]. Recent studies have highlighted the impact of ROS and RNS on neuronal activity, as well as their role in modulating pain perception and transmission [15, 16]. This review focused mainly on the role of ROS relevant to chronic pain.

Over the past 20 years, numerous studies have demonstrated that elevated ROS expression is observed in dorsal root ganglia (DRG), spinal dorsal horn (SDH), or corticolimbic brain regions in nearly all models of chronic pain [16-20], all anatomic sites that contribute to the potentiation and amplification of pain signals [21-26]. Moreover, ROS scavengers can effectively attenuate established chronic pain [27]. It is now generally accepted that ROS play a crucial role in the development and maintenance of central and peripheral sensitization. For example, ROS are involved in promoting phosphorylation levels of N-methyl-D-aspartate (NMDA) receptors, thereby heightening the transmission of excitatory signaling. Meanwhile, they suppress gamma-aminobutyric acid (GABA)-ergic interneurons by reducing synaptic GABA release or diminishing their survival [28-32]. Additionally, ROS can influence nonselective cation channel activity directly through oxidative modification of amino acids and indirectly *via* second messengers [33, 34]. Moreover, ROS induces neuroinflammation by activating glial cells and modulating the expression of anti-inflammatory and pro-inflammatory cytokines in the nervous system [35-40]. The activation of the NOD-like receptor thermal protein domain associated protein 3 (NLRP3) inflammasome may also be triggered by ROS [41].

However, based on the available evidence, the nonselective antioxidants exert limited effects on chronic pain [42, 43]. Possible explanations may be that these antioxidants lack the ability to reverse established oxidative damage. Additionally, inhibition of nociceptive transmission by these antioxidants may be required in specific pain contexts. Moreover, the complexity of ROS regulation is highlighted by the crosstalk between mitochondria and nicotinamide adenine dinucleotide phosphate (NADPH) oxidase, as well as the existence of the ROS-induced ROS release mechanism [44, 45]. All of these factors contribute to the poor ability of antioxidants to address oxidative pain relief. Furthermore, physiological concentrations of ROS are necessary for the homeostasis of the intracellular environment. Therefore, targeting the three main sources of ROS, including mitochondria, NADPH oxidase and peroxisomes, is considered a more promising option for chronic pain treatment rather than directly scavenging free radicals in a broad manner [46]. We reviewed the endogenous molecular signaling pathways and sites involved in the regulation of ROS production, which are implicated in chronic pain management. Additionally, we discussed preclinical evidence aiming to identify new targets and support the development of clinical analgesics.

## 2 TARGETING MITOCHONDRIA FOR CHRONIC PAIN THERAPY

**Fig. (1):**
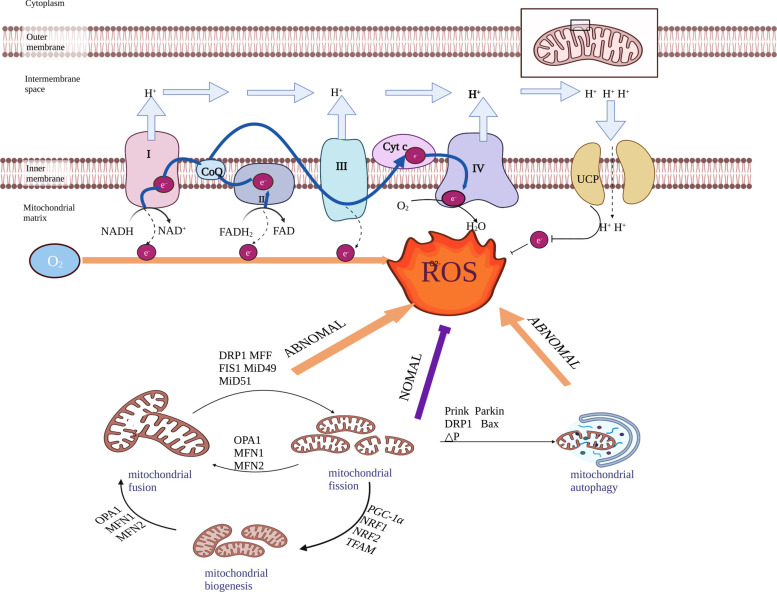
The role of mitochondrial respiratory chain and quality control system in ROS generation. The normal transfer (blue arrows) and leakage process (dotted arrows) of electrons in the mitochondrial respiratory chain. Complex I allows electrons from NADH to enter the mitochondrial respiratory chain [49], Complex II transfers electrons from succinate to CoQ, Complex III transports electrons to cytochrome c and creates a proton gradient [50], Complex IV combines electrons from cytochrome c with oxygen to form water [51], and Complex V synthesizes ATP using the proton gradient [52]. UCP decrease electron leakage and reduce ROS by enhancing normal proton and electron transport. The quality control system of mitochondria maintains their structure and function. If damaged, it disrupts electron flow in the inner membrane, causing more leaked electrons to react with oxygen and generate excessive ROS. (This figure was created with BioRender.com). **Abbreviations**: I: complex I; II: complex II; III: complex III; IV: complex IV; CoQ: coenzyme Q; CytC: cytochrome C; NADH: nicotinamide adenine dinucleotide; FADH_2_: 1,5-dihydroflavin adenine dinucleotide; FAD: flavin adenine dinucleotide; UCP: uncoupling protein; DRP1: dynamic-associated protein 1; MFF: mitochondrial fission factor; FIS1: mitochondrial adaptor fission 1; MiD49: mitochondrial dynamics proteins of 49 kDa; MiD51: mitochondrial dynamics proteins of 51 kDa; OPA1: optic atrophy 1 protein; MFN1: mitofusins1; MFN2: mitofusins2; PGC-1α: peroxisome proliferator-activated receptor-gamma coactivator-1alpha; NRF1: nuclear respiratory factor 1; NRF2: nuclear factor erythroid 2-related factor 2; TFAM: mitochondrial transcription factor A.

Mitochondria are composed of inner and outer membranes, intermembrane space, and matrix [47]. They serve as the primary site for aerobic respiration and are responsible for the production pf approximately 90% of intracellular ROS through oxidative phosphorylation [48]. Under normal conditions, the tricarboxylic acid cycle in the mitochondria matrix produces nicotinamide adenine dinucleotide and 1,5-dihydroflavin adenine dinucleotide (FADH_2_). These molecules donate electrons to the mitochondrial respiratory chain (MRC) on the inner membrane. Approximately 0.2-2% of these electrons leak from the MRC and interact with O_2_ to generate ROS (Fig. **1**) [47-53].

Exposure to exogenous stimulation results in mitochondrial damage, which triggers a sharp increase in ROS generation [54]. ROS can directly attack the MRC, impairing its function and damaging mitochondrial membrane lipids and proteins, leading to membrane receptor inactivation, mitochondrial permeability transition pore opening, increased membrane permeability, and decreased membrane potential. These processes can negatively affect the oxidative phosphorylation function of mitochondria, resulting in further ROS generation. Additionally, due to the lack of protective histones and its imperfect repair system, mitochondrial DNA (mtDNA) is vulnerable to ROS attack, leading to mtDNA mutations. These changes can impact the synthesis of mtDNA-encoded proteins, increasing O_2_**^•−^** generation *via* electron leakage, thereby triggering nucleotide mutations to form a vicious cycle [55]. Overall, mitochondria are highly sensitive to ROS, and their proper functioning is essential for cellular health.

### 2.1 Mitochondrial Oxidative Phosphorylation in Chronic Pain

Complex I-V are components of the MRC. An elegant demonstration of the importance of MRC for chronic pain is that the defect in complex III and IV leads to ROS-dependent neuronal death in the piriform cortex, cingulate cortex, and dentate gyrus [26]. Especially, several complex I-V inhibitors dose (1, 2, 5, and 10 μg)-dependently induce pain-relieving effects in various models of chronic pain [56]. Notably, the time point of intervention, as well as the status of neuronal mitochondria, needs to be considered when applying complex inhibitors. For example, antimycin A, a complex III inhibitor, has a pro-nociceptive effect in normal animals but converts to an antinociceptive effect after paclitaxel treatment, with efficacy only before and during paclitaxel administration [57, 58]. Additionally, rotenone (a complex I inhibitor) and oligomycin (a complex V inhibitor) exhibit intensified nociceptive effects in rats with paclitaxel- or oxaliplatin-induced neuropathic pain, potentially attributed to the variations in the experimental models employed [59]. In addition, Hamblin *et al.* proposed that photobiotherapy specifically targets complex IV to reduce ROS levels in stressed tissues and primary cultured cortical neurons [60, 61]. In a complete Freund's adjuvant (CFA)-induced inflammatory pain model, researchers were convinced that the analgesic effect of photobiotherapy is associated with this modulation [62]. Moreover, a clinical study suggested that arginine increases platelet mitochondrial complex IV and V synthase activity and reduces ROS levels, thereby alleviating pain in children with sickle cell disease [63]. This finding may provide insights for the treatment of various chronic pain conditions involving mitochondria.

Coenzyme Q (CoQ) 10 is a key carrier for transferring electrons in the MRC to produce ATP. It exists in three interchangeable forms: ubiquinone, ubisemiquinone radical, and ubiquinol. Among them, the ubisemiquinone radical serves as the primary source of mitochondrial superoxide radicals, and a reduction in its level or the destruction of its binding site increases superoxide production by affecting reverse electron transport [49, 50]. CoQ10 is effective in alleviating fibromyalgia, trigeminal neuralgia, and statin-induced myalgia due to its capacity to reduce ROS stress levels [64-66]. In a carrageenan-induced inflammatory pain model, the combination of idebenone (CoQ10 analog) and cyclodextrin, which improved the bioavailability of idebenone, was significantly reduced the ROS levels in the spinal cord, thereby inhibiting thermal hyperalgesia and paw edema [67, 68]. In addition, plasma CoQ10 levels are a reliable marker reflecting the degree of oxidative stress in the human body [69, 70]. Considering that CoQ is widely used as an anti-aging health supplement and for alleviating conditions linked to mitochondrial dysfunction, the findings from the aforementioned preclinical study suggest that CoQ may soon be tested in clinical trials to investigate its protective effects against chronic pain [71].

Uncoupling protein (UCP) is a mitochondrial inner membrane protein, and UCP2-5 reduces the mitochondrial membrane potential (MMP) by uncoupling survival mechanisms, thus preventing mitochondria from generating excess ROS [72]. Upregulation of UCP3 inhibits oxidative stress to protect rats with occlusal interference far from muscle pain caused by masseter damage [73, 74].

Carbonic anhydrase (CA) is a zinc-containing metalloenzyme that catalyzes the reversible hydration reaction of CO_2_ to form bicarbonate and protons. Members of its family are localized to special cellular and subcellular sites and show tissue-specific expression. Among them, CAVA and CAVB are localized in mitochondria and are involved in the regulation of respiration rate and ROS production [75]. Excess mitochondrial CA leads to the production of many electron donors that generate a high MMP by transporting protons across the inner mitochondrial membrane, which can inhibit electron transport in complex III and prolong the half-life of CoQ radical intermediates, subsequently increasing the chance of O_2_**^• −^** being reduced to superoxide [76]. Micheli *et al.* demonstrated that mitochondrial CAVA and CAVB inhibitors 5b and 5d counteract maladaptive plasticity in glial cells and reduce oxidative stress injury due to mitochondrial dysfunction in the central nervous system (CNS). Nociceptive hypersensitivity was well suppressed in experimental animals after acute and subchronic treatment, suggesting the therapeutic and protective significance of CA inhibitors for paclitaxel-induced neuropathy [75].

FAM173B, a mitochondrial lysine-specific protein methyltransferase, is primarily localized within the mitochondrial cristae [77]. Deficiency of FAM173B reduces both maximal respiration and proton leakage by 50%, leading to decreased ROS production [78]. *In vitro*, human FAM173B promotes ROS formation in primary sensory neurons. *In vivo*, high expression of FAM173B induced by carrageenan injection increases ROS levels in spinal sensory neurons, and intrathecal injection of FAM173B antisense oligodeoxynucleotide attenuates the development of inflammatory pain. These results fully confirm that inflammatory-induced hypersensitivity depends on the regulation of ROS by FAM173B in sensory neurons. Similar results were observed in a model of neuropathic pain (NP) induced by spared nerve injury (SNI). In addition, the chronic pain-promoting effect of FAM173B is attributed to its methyltransferase activity [77], because the methyltransferase-deficient mutant human FAM173B-D94A does not extend carrageenan-induced pain behavior [77]. Finally, whole-genome sequencing in an Icelandic population showed that targeting FAM173B seems to be available, as FAM173B-deficient populations were healthy [79]. Knockout of this “pain gene” may be the fundamental solution for the pain problem that plagues humans.

### 2.2 Mitochondrial Quality Control Systems in Chronic Pain

Several findings from clinical research and preclinical studies from many laboratories have demonstrated that mitochondrial dysfunction may play a critical role in multiple forms of chronic pain, including NP, inflammatory pain, and morphine tolerance [19, 57, 80, 81]. For example, chemotherapy drug-induced mitochondrial dysfunction in both A- and C-fiber nociceptors of the sciatic nerve is associated with concurrent pain behaviors [57]. Mitochondria have evolved into various quality control systems, specifically mitochondrial dynamics, mitochondrial biogenesis, and mitophagy (Fig. **1**) [82]. The relationships among them are bidirectional, indicating that they both change and are changed by the form-function dynamics of mitochondria. In particular, inefficient autophagy may contribute to abnormal mitochondrial function and ROS accumulation [83]. This vicious cycle of abnormal mitochondrial quality control and biomolecule regulation provides a feedforward mechanism leading to further accumulation of mitochondrial ROS. Therefore, focusing on mitochondrial quality control might be a useful approach for chronic pain treatment.

#### 2.2.1 Mitochondrial Fission/Fusion

Mitochondria are highly active organelles that maintain their balance by segregating injured modules by fission and exchanging contents among viable mitochondria *via* fusion. The proteins involved in these processes are dynamic-associated protein 1 (DRP1) and mitochondrial adaptor fission 1 (FIS1), which aid in fission, and mitofusion1/2 and optic atrophy 1 protein (OPA1), which regulate fusion [84].

DRP1 is widely expressed in the CNS, particularly in the “pain-related” laminae I and II of SDH [85]. Primarily localized in the cytoplasm, DRP1 is recruited to the outer mitochondrial membrane by FIS1, Mff, MiD49 and MiD51 molecules, enriched in potential mitochondrial fission sites and forming finger-loop structures around the mitochondria, which contract and ultimately split the mitochondria in a GTPase-dependent manner [84]. DRP1-mediated excess mitochondrial splitting or fragmentation was shown to be a key factor in pathological ROS generation [86], and the DRP1-mitochondrial fragment-ROS axis is involved in the mechanism of neuronal injury in the prefrontal cortex [87], thus, it is not surprising that this protein has been implicated in nociception. Indeed, the absence of DRP1 reverses remifentanil-induced hyperalgesia by downregulating spinal NMDAR expression through the DRP1-mitochondria-ROS pathway [88]. In addition, a clinical report of patients with DRP1 gene mutations exhibiting pain insensitivity also confirmed that normal DRP1 expression may be associated with chronic pain [89]. Wrapping gp120-infused oxidized regenerated cellulose around the sciatic nerve caused NP, resulting in increased DRP1 expression in the SDH [90]. Similarly, the analgesic effect of MitoTEMPO may be partly attributed to its modulation of DRP1, as it is able to reverse the abnormally high expression of DRP1 in the DRG of chronic constriction injury (CCI) rats [91].

Moreover, DRP1 function and expression are regulated by various posttranslational modifications (*e.g*., phosphorylation, nitrosylation, sumoylation, *etc*.) [92]. 2-Bromopalmitate blocks the palmitoylation of DRP1 by inhibiting the activity of the palmitoyltransferase ZDHHC13. It was shown that 2-Bromopalmitate treatment reduces DRP1 expression, restores the balance of mitochondrial division and fusion in the spinal cord, and reduces proinflammatory cytokine expression and ROS production, and these changes were accompanied by an increase in oxaliplatin- and CFA-induced paw withdrawal threshold in rats after 2-Bromopalmitate treatment [93, 94]. These results may partially reveal the effect of DRP1 palmitoylation on oxidative stress and chronic pain.

Unexpectedly, in another study, the investigators concluded that upregulation of DRP1 in the SDH may be beneficial for pain symptoms in experimental mice, as intrathecal injection of adeno-associated virus-induced high DRP1 expression and successfully alleviated SNI-induced pain behavior. They then explored the regulation of DRP1 on mitochondrial function and ROS in the context of pain. Based on transmission electron microscopy analysis of mitochondrial ultrastructure, they concluded that overexpression of DRP1 significantly ameliorated SNI-induced changes in vesicle parameters. In addition, DRP1-mediated ROS expression is bidirectional, as knockdown or overexpression of DRP1 up- or downregulates ROS expression levels, respectively [95].

#### 2.2.2 Mitochondrial Biogenesis

Mitochondrial biogenesis is the process by which new mitochondria are generated from existing mitochondria, and it has been proven to promote the recovery of mitochondrial function. This process is mainly regulated by peroxisome proliferator-activated receptor-gamma coactivator-1alpha (PGC-1α) and mitochondrial transcription factor A (TFAM). The former coordinates mitochondrial biogenesis by acting as a coactivator of multiple transcription factors, and the latter is one of the major transcription factors involved in the induction and coordination of mitochondrial genome expression.

PGC-1α activates nuclear respiratory factor 1 (NRF1) and NRF2, which subsequently regulate TFAM translocation into the mitochondrial matrix and stimulate mitochondrial DNA replication and gene expression [96]. Its reduction is responsible for the extensive neuronal loss in the cingulate gyrus and frontal cortex [97]. In neuronal B35 cells, recombinant PGC-1α prevents the increased expression of ROS under tumor necrosis factor α (TNFα) application. In morphine-tolerant mice, PGC-1α can reduce ROS levels and increase mechanical and thermal hypersensitivity [17]. PGC-1α plays an inhibitory role in nociception, as supported by a study demonstrating that intrathecal injection of ZLN005 (a PGC-1α activator) markedly reduced ROS generation and alleviated pain behaviors in CCI mice [98]. Recently, several studies have shown that PGC-1α also has a protective effect against sciatic nerve ligation (SNL) and paclitaxel-induced NP [99, 100].

NRF2 is a transcription factor that regulates the expression of genes involved in antioxidant defense and detoxification. Its activation proviedes benefits in chronic pain primarily through the alleviation of ROS-associated pathological processes [101,102]. Recently, it was found that NRF2 can directly regulate PGC-1α expression and participate in mitochondrial biogenesis [103]. The therapeutic potential of strategies targeting NRF2 was tested in CCI rats by intrathecal injections of RTA-408 (an NRF2 activator). RTA-408 restored mitochondrial bioenergetics and suppressed oxidative stress, which is the key mechanism of the analgesic effect of RTA-408 [98]. Unsurprisingly, pretreatment with either trigonelline (an NRF2 inhibitor) or SR-18292 (a PGC-1α inhibitor) completely prevented the alleviating effect of RTA-408 [98]. Approaches targeting the newly discovered role of NRF2 to develop strategies that target mitochondrial biogenesis are encouraging therapeutic options for chronic pain.

Monoamine oxidases (MAOs) includes two isoforms. MAO-A is responsible for the oxidative deamination of 5-hydroxytryptamine (5-HT) and norepinephrine while MAO-B has a major affinity for benzylamine and phenylethylamine. It is anchored to the outer mitochondrial membrane by a transmembrane helical structure located in the carboxy-terminal structural domain and is considered to be the main producer of mitochondrial ROS [104]. Studies have shown that KDS2010 (an MAO-B inhibitor) exerts similar antinociceptive effects as the ROS scavenger phenyl-N-tert-butylnitrone (PBN) in paclitaxel-induced NP. Furthermore, researchers have found that KDS2010 partially restores the weakened inhibitory GABA synaptic transmission in the spinal cord of mice treated with paclitaxel. Additionally, KDS2010 exhibits a similar effect as PBN in terms of restoring the frequency of spontaneous inhibitory postsynaptic currents (sIPSCs), suggesting that KDS2010 may restore sIPSCs frequency by targeting ROS. These findings provide evidence supporting the hypothesis that KDS2010 acts on ROS-GABA synaptic transmission to alleviate the paclitaxel-induced NP [105]. Furthermore, it has been found that elevated levels of 5-HT and norepinephrine in the thalamus and prefrontal cortex have shown promising results in relieving symptoms of fibromyalgia [106]. These effects could be attributed, at least in part, to the regulation of PGC-1α. Evidence suggests that SR-18292 (a PGC-1α inhibitor) partially abolished the analgesic effects of lasmiditan (a 5-HT agonist) and formoterol (a β2-adrenoreceptor agonist) [99, 100]. All these results indicate that the potential efficacy of MAOs in treating chronic pain is related to ROS generation *via* enzymatic reactions or *via* alterations in the levels of MAO substrates. It is worth mentioning that activation of β-adrenergic receptors in SDH neurons increases the cAMP contents in these neurons [107]. The elevation of cAMP triggers a cascade of reactions that activate cAMP-response element-binding protein (CREB) in SDH neurons [108]. This activation is closely associated with the development of cancer-induced bone pain [109]. Additionally, CREB play a significant role in the transmission of HIVgp120-induced neuropathic pain through the cytoplasmic polyadenylation element-binding protein-ROS-CREB binding protein signaling pathway [110]. Furthermore, the U.S. Food and Drug Administration has approved 5-HT receptor agonists and β2-adrenoreceptor agonists for the clinical treatment of migraine and asthma, respectively [111, 112]. These drugs may quickly be entered into clinical trials investigating their protective effects against chronic pain.

#### 2.2.3 Mitophagy

Mitophagy is the targeted phagocytosis of damaged mitochondria by the cellular autophagic apparatus [113]. Parkin protein is recruited to damaged mitochondria and acts as a ubiquitin ligase to mediate mitochondrial autophagy.

P53 is a well-known tumor suppressor gene. Recent studies have found that in isolated cortical neurons, the p53 protein can negatively regulate the expression level of the parkin protein and subsequently impact the autophagy processes [114]. Next, Yamashita *et al.* investigated this regulatory mechanism in the context of diabetic NP, and they showed that the levels of p53 in the DRG were significantly higher in streptozotocin (STZ)-treated rats than in wild-type animals. Rescue of low parkin expression by pifithrin-µ (a p53 inhibitor) was found to suppress ROS accumulation and attenuated mitochondrial dysfunction in DRG neurons, as well as to reduce pain hypersensitivity after STZ treatment [115]. In another study, Krukowski *et al.* linked CINP to p53-mediated mitochondrial destruction. They showed that the alteration in mitochondrial morphology caused by blocking the abnormal accumulation of p53 with pifithrin-µ (an inhibitor of mitochondrial binding of p53) prevents the prevention of mechanical allodynia in mice [116, 117]. In addition, pifithrin-µ interferes with the binding of HSP70 to apoptotic protease activating factor-1 and inhibits the formation of the HSP70/p53 complex, resulting in the death of tumor cells [118]. Therefore, the combination of pifithrin-µ with chemotherapeutics may be the best option for certain cancers.

Mammalian ste20-like kinase 1 (Mst1) is a member of the Ser/Thr protein kinase family. It has been demonstrated that Mst1 regulates parkin-dependent mitochondrial autophagy by Mst1 in the myocardium [119]. Recently, Huang *et al.* investigated the role of Mst1 in CCI mice and showed that MsT1 expression is elevated in the sciatic nerve after CCI and that small interfering RNA (siRNA)-mediated disruption of Mst1 suppresses established pain. Finally, they confirmed that Mst1 attenuates the parkin recruitment from the cytoplasm to the mitochondria in RSC96 cells *in vitro* [120].

#### 2.2.4 Mitochondrial Protection

Sirtuins (SIRT) 1-7, a family of NAD^+^-dependent deacetylases, have been recognized for their ability to protect mitochondria by modulating oxidative stress [121]. Among them, SIRT1 and SIRT3 have received much attention for their role in chronic pain.

SIRT1 localizes in the nucleus and mediates deacetylation of PGC-1α, a key pathway that alters PGC-1α activity [122]. Activation of the SIRT1/PGC-1α signaling pathway has been associated with the dysfunction of amygdala parvalbumin interneurons [123]. Several findings have proven that activation of this pathway can reverse the characteristic changes related to the pain of several etiologies, including paclitaxel- and SNL-induced NP as well as low back pain [124-126].

SIRT3 is mainly localized in the inner mitochondrial membrane [127]. Indeed, SIRT3-deficient hippocampal and striatal neuronal cells exhibit significantly increased mitochondrial ROS, which are restored to physiological levels when SIRT3 is overexpressed [128]. These results imply the possible potential analgesic properties of SIRT3. In a diabetes model, Zhou *et al.* discovered that the expression of SIRT3 in the SDH was reduced on day 21 after STZ injection and found that this change was responsible for diabetic NP, as local overexpression by delivery of the lentiviral vector into the SDH suppressed established pain. To confirm the role of SIRT3 in pain mechanisms, the authors induced downregulation of SIRT3 in the intact rat SDH, which was sufficient to elicit pain-related behaviors [129]. The analgesic effect of SIRT3 is attributed to its deacetylase activity. Specifically, it reduces the acetylation of cyclophilin D and forkhead homeobox type O 3a, providing relief from pain symptoms [19, 129]. Additionally, SIRT3 undergoes posttranslational modifications involved in maintaining the pain state. Carbonylation of SIRT3 caused by lipid peroxidation decreases its deacetylase activity and disrupts metabolic balance, thereby intensifying the sensation of pain. In a carrageenan-induced inflammatory pain model, elevated levels of 4-hydroxynonenal (a product of lipid peroxidation) and SIRT3 carbonylation were observed in the spinal cord. Inhibition of lipid peroxidation resulted in reduced SIRT3 carbonylation and alleviation of pain sensitization [130]. In conclusion, these amplified automatic feedback loops regulating SIRT3 activity and the positive feedback signaling enhancing oxidative damage suggest that restoring mitochondrial SIRT3 function and upstream and downstream pathways may be an innovative approach for chronic pain treatment.

Adenosine monophosphate-activated protein kinase (AMPK) is a widely distributed serine/threonine protein kinase that exists as a heterotrimeric complex in neurons and glial cells of the central nervous system (CNS) [131]. It is a well-known inducer of autophagy. Activation of AMPK can lead to the phosphorylation and activation of Unc-51-like kinase 1, which in turn phosphorylates and activates parkin. This activation of parkin promotes the tagging of damaged mitochondria for degradation by autophagosomes. As a key regulator of mitochondrial homeostasis, AMPK also influences mitochondrial quality control by impacting mitochondrial dynamics and biogenesis [132]. Moreover, AMPK activation has been shown to suppress nuclear factor-kappa B (NF-κB) signaling through multiple mechanisms [133-135]. By inhibiting IκB kinase activity and preventing p65 subunit nuclear translocation, AMPK reduces NF-κB dependent gene transcription [135, 136]. This NF-κB inhibition can contribute to decreased ROS production, as NF-κB can induce the expression of ROS-generating enzymes [137]. As expected, the activation of NF-κB in microglia contributes to the initiation of SNI-induced pain [138]. Maixner *et al.* showed that in a pSNL model, AMPK activity in the SDH is reduced in the late phase of NP, *i.e*., 10 days postoperatively. This alteration is responsible for NP, as pharmacological activation of AMPK activity inhibits established pain. To validate the role of AMPK in the pain mechanism, the authors induced a downregulation of AMPKα in the SDH of intact rats and found that it was adequate to elicit pain-related behaviors. The authors then further clarified that AMPKα1 is the specific AMPKα isoform associated with nociceptive processing, as AMPKα1 knockout mice exhibited similar changes in pain behavior as rats with siRNA-mediated knockdown of AMPKα [139]. The pain induced by aberrant AMPKα1 expression is linked to AMPKα-mediated regulation of ROS, as the ROS scavenger PBN was found to attenuate the heightened frequencies and amplitudes of miniature excitatory postsynaptic currents (mEPSCs) in SDH induced by AMPKα1 deficiency, as well as behavioral hypersensitivity [140]. Similarly, AICAR (an AMPK activator) attenuates DRP1-mediated mitochondrial fission and blocks NLRP3 inflammasome-mediated neuroinflammation, which in turn lessens cancer-induced bone pain [141,142]. In addition, metformin (an activator of AMPK) decreases ROS production in the sciatic nerve of diabetic mice, thereby alleviating mechanical pain sensitivity. This beneficial effect may be related to the regulation of autophagy, as the expression of Beclin-1 and LC3B was found to be significantly lower in metformin-treated diabetic mice [143]. The ability of AMPK to inhibit chronic pain-induced oxidative stress affords an additional mechanism for the protective effects of metformin in diabetic patients. Consistent with this hypothesis, compound C (an AMPK blocker) attenuates mitophagy, which in turn increases the accumulation of ROS, worsening outcomes following spinal cord injury (SCI) [144].

## 3 TARGETING NADPH OXIDASE FOR CHRONIC PAIN THERAPY

**Fig. (2):**
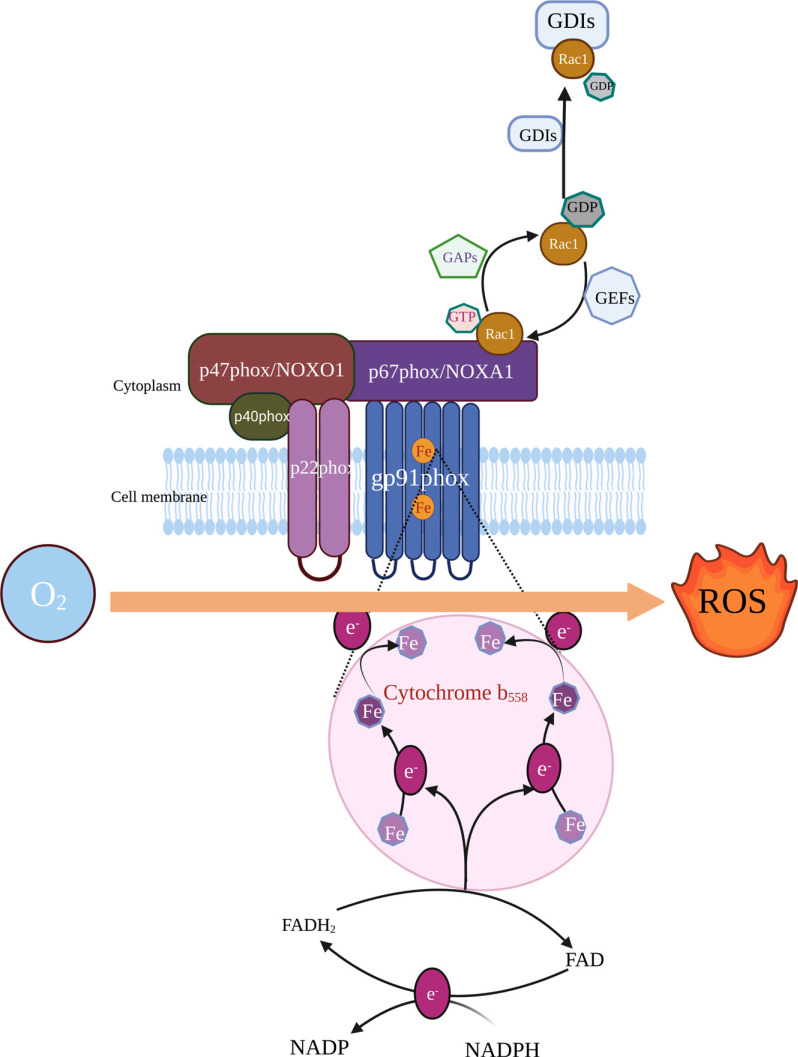
The role of NADPH oxidase family in ROS generation. Rac1 is activated when it binds to GTP and is inactivated when it binds to GDP. Guanine nucleotide exchange factor (GEF) is responsible for the conversion of GDP to GTP, and GTP-activating proteins (GAPs) promote Rac1 proteolysis. Guanine nucleotide dissociation inhibitors (GDIs) block the activation pathway of Rac1 [173, 174]. p67phox, p40phox and activated Rac1 are assembled and then induced to bind and translocate to the cell membrane by phosphorylated p47phox, where they combine with the complex composed of p22phox and gp91phox in the plasma membrane to form the active form of nicotinamide adenine dinucleotide phosphate (NADPH) oxidase. The NOX protein contains binding sites for NADPH, flavin adenine dinucleotide (FAD) and heme. The NADPH substrate donates two electrons, and these electrons are transferred to FAD, which is reduced to FADH_2_. FADH_2_ transfers the first electron to the cytochrome proximal ferrous to form the semiquinone-based form of FAD, which is rapidly transferred to the distal heme to form ROS with the molecular oxygen acceptor. The second electron carried by the semiquinone-based FAD combines with oxygen molecules in the same way to form ROS [146]. (This figure was created with BioRender.com).

The NADPH oxidase family, originally described in the context of neutrophils and macrophages, constitutes an important line of defense for host immune defense during respiratory outbreaks [145]. By transferring cytoplasmic NADPH-derived electrons to O_2_, NADPH oxidase generates ROS and transport them into the cytosol through aquaporin channels. The NADPH oxidase family members possesses six transmembrane-spanning structural domains. The catalytic subunit gp91phox (later also called NOX2) is the most important part for its role, as it contains the redox center for oxidation of NADPH and reduction of O_2_, as well as the binding sites for NADPH, flavin adenine dinucleotide (FAD) and heme (Fig. **2**) [146]. In recent years, seven homologs of NOX2 have been successively identified in different cells, named the NOX family, of which NOX1, NOX2 and NOX4 are closely associated with chronic pain.

### 3.1 NOX Isoforms in Chronic Pain

NOX1 is distributed in neurons, macrophages and glial cells in the CNS and DRG [147-149]. A previous experiment using NOX1 knockout mice confirmed that NOX1 plays a pronociceptive role in morphine tolerance. Basically, it facilitates the pain produced by morphine injection [148]. High levels of NOX1 mRNA were detected in the DRG, and pharmacological inhibition or genetic deletion of NOX1 reduces pain behavior in mice with inflammatory pain [149, 150]. NOX1-derived ROS activate and induce the translocation of protein kinase C (PKC) ε to increase transient receptor potential vanilloid 1 activity in DRG neurons, promote ERK1/2-NF-κB signaling and participate in glial cell activation in the DRG and CNS [149, 151]. Furthermore, the oxidation of NMDA in the prefrontal cortex is similarly facilitated by ROS produced by NOX1 [152]. These processes have a well-recognized role in chronic pain [153].

NOX2 is expressed in spinal microglia and neurons, DRG-stationary or recruited macrophages, and damaged peripheral nerves [16, 22, 37, 154]. Upregulated expression of NOX2 mRNA and correspondingly high levels of ROS have been reported in the spinal cords of mice with sciatic nerve transection (SNT), SCI and cancer-induced bone pain, as well as in the DRG of SNI mice, and global knockout or pharmacological inhibition of NOX2 significantly suppresses established pain [16, 37, 154, 155]. Spinal NOX2-derived ROS play a role in the development of NP by affecting synaptic plasticity, particularly long-term potentiation.This is achieved through increases in GluN2B (NMDA receptor) phosphorylation and NF-κB p65 level, as well as an increase in the spontaneous excitatory postsynaptic currents (sEPSCs) frequency in laminar II neurons [22]. It also increases M1 polarization of spinal microglia/macrophages after SCI, which may involve regulation of the IL-10/microRNA155 (miR155) signaling pathway, as NOX2 depletion leads to downregulation of miR155, which may be due to increased IL-10 signaling [155]. Peripheral NOX2-dependent mechanisms may be more conducive to the maintenance of NP. In DRG, NOX2 promotes TNFα-related signaling interactions between macrophages and neurons and subsequently activates transcription factor 3 expression following peripheral nerve injury [154]. Interestingly, NOX2 deficiency does not affect macrophage recruitment to the injured DRG [154]. As these studies were conducted in a global intervention, there is still no definitive evidence regarding whether changes in the spinal cord and DRG are influenced by NOX2-dependent alterations in macrophage function at the site of injury. NOX2ds-tat did not exert similar anti-injury effects in SCI mice after pSNI [155, 156], which may have been related to using different animal models. Identifying the optimal therapeutic window is important for the control of NOX2 signaling kinetics in chronic pain, as inhibition of NOX2 by shRNA or NOX2ds-tat was found to prevent high-frequency stimulation-induced persistent mirror pain, whereas NOX2ds-tat posttreatment was ineffective in the same animals [22]. Unlike NOX1, NOX2 in macrophages does not contribute to inflammatory pain [157]. NOX2 also contributes to morphine tolerance. NOX2 knockout prevents the development of morphine tolerance in the late phase by increasing the activation of anti-inflammatory factors (IL4 and IL10) and by decreasing the formation of proinflammatory cytokines (TNFα and IL1β) [158]. Notably, complete loss of NOX2 function leads to chronic granulomatous disease [159], which may be a safety risk that must be taken with NOX2 pharmacological inhibition.

NOX4 is inducibly expressed by microglia in spinal and nonpeptidergic neurons in the DRG, as well as in injured peripheral nerves [20, 23]. It is closely associated with pain signaling in rodent models, and pain behavior is significantly attenuated in NOX4-deficient animals [20, 23]. NOX4 contributes more to NP but less to inflammatory pain and acts mainly at the lesion site in peripheral nerves [20, 160]. NOX4 knockdown was found to abolish SNI-induced degradation of the peripheral myelin proteins MPZ and PMP22, demonstrating that NOX4 maintains NP through demyelination, as evidenced by morphological analysis of injured nerves [20]. A follow-up study by the same group identified the small calcium-binding protein S100A4 as the target of NOX4-mediated oxidation [161]. NOX4 appears to play a role in both the subacute and late stages of NP, as NOX4-deficient mice exhibit a similar degree of mechanical hypersensitivity as WT mice in the first 7 days after CCI or SNI [20]. However, another study showed that targeting NOX4 should occur at an ultra-early stage after peripheral nerve injury. Post-injury treatment (1-21 days after CCI) with GKT136901 (a NOX1/4 inhibitor), which has been shown to be effective for systemic NOX4 inhibition in atherosclerotic mice [162], did not result in an improvement in nociceptive behavior [160]. In addition, the same study revealed that NOX4 deletion benefits pain symptoms in mice through inflammation control after injury [160]. Together with NOX2, MOX4 constitutes the main source of ROS in the sciatic nerve in diabetic mice [163]. Finally, the result is that the downregulation of NOX4 inhibits GABAA-γ2 and NMDAR expression in the SDH to alleviate bone cancer pain, compensating for the contribution of NOX4 to central sensitization [23, 164]. In conclusion, NOX4 is an excellent therapeutic candidate for chronic pain, but the therapeutic window for pharmacological intervention requires further exploration.

### 3.2 Novel Strategies Targeting the NADPH Oxidase Family and Their Therapeutic Potential in Chronic Pain

Since NADPH oxidase is the only known family of enzymes with the sole function of producing ROS, it may represent a major disease mechanism for chronic pain and a target for mechanism-based defense against oxidative damage. NOX2/NADPH oxidase activation requires six proteins, namely, NOX2, p22phox, p67phox, p40phox, p47phox, and activated Rac1 (Fig. **2**) [165]. Similarly, NOX1 complex activation requires the formation of a complex between NOXO1 and NOXA1. During this process, NOXO1 acts as a scaffold for binding NOXA1 and p22phox to enhance the efficiency of the NOXA1-NOX1 interaction. The activity of NOX4 appears to correlate only with enzyme expression [166]. Despite abundant clinical and preclinical evidence for the critical role of different NOX isoforms in chronic pain, the development of their inhibitors is lagging behind [167]. Interestingly, *in vitro* studies have shown that p47phox and p67phox can assemble and activate the NOX1 and NOX3 complexes in place of NOXO1 and NOXA1, suggesting a common mechanism for the activation of NADPH oxidase family members. This may also explain the failure of specific inhibitors [165, 168, 169]. Strategies for attenuating NOX-derived ROS generation other than inhibition of NOX alone are needed to prevent the development of chronic pain. We realized that the absence of expression of any NOX2 complex components results in a chronic granulomatous disease phenotype [170, 171]. This fact inspired us to develop strategies to control NADPH oxidases, including a) modulating the expression of other NOX complex components and b) interfering with upstream signaling molecules [172].

#### 3.2.1 Therapeutic Potential of Other NOX Complex Subunits in Chronic Pain

Rac1 is a Rho GTPase and is converted from an active state to an inactive state through the G protein cycle (Fig. **2**) [173, 174]. In addition to acting as a direct participant, it can induce the membrane translocation of p47phox and p67phox during the activation of NADPH oxidase. In this process, the expression level of Rac1 plays a pivotal role than the integrity of the insertion domain [175]. Not surprisingly, NSC23766, a Rac1 inhibitor, was found to exert potent analgesic effects in the rat model of burn injury and paclitaxel-induced NP [176, 177].

Apurinic/apyrimidinic endonuclease 1/redox effector factor-1 (APE1/Ref-1) is widely expressed in mammalian cells and participates in DNA base excision repair and redox-sensitive transcriptional regulation. It may relocalize relying on tissue or cell type, metabolic status, and stress condition, indicating that their subcellular localization and trafficking are important for regulating function [178]. Specifically, in human umbilical vein endothelial cells, APE1/Ref-1 inhibits Rac1-induced H_2_O_2_ elevation by their extranuclear function [179]. Due to the ubiquitous distribution of APE1/Ref-1 and Rac1, their functional interaction may also be related to redox biology in other cell types. Zaky *et al.* reported that oxidative stress is regulated *via* APE1/Ref-1 in the chronic pain background. They showed that under inflammatory conditions, APE1/Ref-1 expression decreases and its nuclear accumulation increases [180]. Cotreatment with E3330 (an APE1 inhibitor) and CFA induces cytosolic translocation of APE1/Ref-1 and restores the total tissue antioxidant capacity to normal levels in the spinal cord, thereby inhibiting mechanical allodynia in mice. All these results suggested that both the expression and subcellular localization of APE1/Ref-1 for resisting oxidative stress underlying inflammatory pain [180]. Interestingly, the effect of APE1/Ref-1 in suppressing H_2_O_2_ is due to its ability to reduce H_2_O_2_ production, as overexpression of APE1/Ref-1 does not reverse the increase in cellular H_2_O_2_ levels caused by exogenous H_2_O_2_ [179]. As key upstream factors in cellular defenses against oxidative stress, APE1/Ref-1 signaling has an innate advantage in alleviating pain.

Ryanodine receptors (RyRs) are intracellular calcium channels expressed in neural tissue that mediate the rapid release of calcium ions from the endoplasmic reticulum (ER) and sarcoplasmic reticulum [181], resulting in Rac1 activation [182]. In an SCI model, genetic deletion of RyR induced the expected suppression of mechanical allodynia by inhibiting NOX2-derived ROS levels and the release of inflammatory factors in the spinal cord [183]. Interestingly, in another experiment, Wilson *et al.* verified the mechanism by which neuronal ROS regulate Rac1 activity through RyR-mediated Ca^2+^ release [184]. A feedforward loop between ROS and Rac1 is formed, and these interactions generate both an amplified automatic feedback loop that modulates RyR activity and a positive feedback loop that amplifies ROS signaling and exacerbates oxidative damage. Unfortunately, direct evidence for a feedforward mechanism in the context of chronic pain is lacking. In addition, RyR-mediated Ca^2+^ release triggers mitochondrial fragmentation, leading to mitochondrial ROS generation [185]. Godai *et al.* assessed the significance of RyR-mediated regulation of mitochondrial ROS in HIV-related NP. The authors revealed that RyR and mitochondrial superoxide levels were increased in the SDH, whereas intrathecal injection of dantrolene (a RyR antagonist) conspicuously reduced ROS levels and increased the pain threshold in rats [186].

Caveolin-1 (Cav-1) functions as a specific regulator in biological processes such as signal transduction and cell regulation beyond its role as a major structural protein for caveolae formation [187]. Specifically, it is an important mediator of Rac1/NOX-derived ROS generation [188]. Researchers quantified the expression of Rac1 and NOX2 in STZ-induced diabetes rats to verify the role of this regulatory mechanism in chronic pain. They observed that the proteins were significantly upregulated compared to controls. Subcutaneous injection of a Cav-1 inhibitor reduced NOX2 expression, thereby inhibiting NMDA receptor activation and dramatically reversing established mechanical and thermal hyperalgesia [189].p47phox acts as an important adaptor protein conveying the cytosolic complex (consisting of p47phox-p67phox-p40phox) to the plasma membrane to complete docking with cytochrome b558 (comprising gp91phox and p22phox) during NADPH oxidase activation [190]. Indeed, in the streptozotocin-induced type 1 diabetes model, p47phox and gp91phox are upregulated in the spinal cord and correlated with painful behavior [191]. Genetic approaches disrupting p47phox result in a complete attenuation of morphine tolerance [158]. In addition, p47phox might be the major mediator of C-C motif chemokine ligand 2 (CCL2)-induced oxidative stress and hyperalgesia. For the genetic polymorphism of ncf1 coding for p47phox [192], Dark Agouti rats exhibited less painful behavior in response to CCL2 than Wistar rats [157]. Intra-articular injection with p47phox siRNA-loaded poly (D, L-lactic-co-glycolic acid) nanoparticles showed fruitful internalization and significantly attenuated the oxidative stress in osteoarthritis mice cartilage [193]. NOX2ds-tat specifically targets the p47phox subunit and, as expected, their binding blocks the assembly and activation of the NOX2 complex [172], thus exerting a powerful anti-injurious effect [22, 155].

p22phox contributes to chronic pain processing. Quantitative real-time polymerase chain reaction showed that p22phox mRNA in the DRG of SNI animals was upregulated 3-14 days after surgery [154]. Studies have shown that p22phox is upregulated in the sciatic nerve of diabetic rats. This change is associated with increased ROS production and oxidative DNA damage [194]. Plasma membrane translocation of NOXO1 in nociceptive neurons induces ROS production, which develops into inflammatory pain [195]. Some preclinical studies have also indicated that curcumin and jinmaitong exert favorable analgesic effects [196]. Their ability to inhibit the NADPH oxidase subunits p22phox, p47phox and gp91phox provides an alternative mechanism for their protective effects against chronic pain [191, 194].

At present, research on the functional subunit of NOX mainly focuses on cardiovascular and cerebrovascular diseases, and research on chronic pain is still relatively rare. The physiological and pathological functions of NOX regulatory subunits in the context of chronic pain and their establishment as targets for regulating pain require more direct evidence. Moreover, natural and traditional Chinese medicine compounds generally exert pleiotropic and direct antioxidant effects. Thus, off-target screening of these compounds is required before on-target *in vivo* proof-of-concept studies.

#### 3.2.2 Analgesic Potential of Targeting Pathways of NOX-derived ROS Generation

Hydrogen voltage-gated channel 1 (Hv1) regulates NOX activity by maintaining the balance of H^+^ current and e-current produced by NOX in the phagocyte membrane [197]. Recently, Peng *et al.* investigated the regulation of NOX2 by Hv1 during NP [198]. They found that the Hv1 proton channel is functionally expressed in spinal microglia and is upregulated after SNT and common peroneal nerve ligation, paralleling a critical time window for NP development. Pain behavior in Hv1 KO mice was significantly reduced compared to that in WT mice. These experimental results confirm the role of Hv1 in the mechanism of NP. Next, the authors quantified NOX2 expression and found that it was significantly lower in the spinal cords of Hv1-KO mice than in those of wild-type animals, suggesting a causal relationship. Importantly, the administration of ROS scavengers reduced mechanical allodynia induced by peripheral nerve injury in intact mice but had no effect on Hv1-KO mice. These results convincingly suggest that the regulation of NOX2/ROS by Hv-1 contributes to NP [198]. Moreover, the benefits of Hv1 in reducing inflammatory pain and morphine-induced hyperalgesia and tolerance were also confirmed by pharmacological blockade of Hv1 or knockdown of the HV1 gene [199]. Interestingly, studies have confirmed that Hv1 promotes cancer cell growth and metastasis [200]. Based on these results, it is a good candidate target for relieving pain, especially cancer pain.

The transient receptor potential ankyrin 1 (TRPA1) channel, a nonselective cation channel expressed in the terminals of sensory nerves, is susceptible to modulation by ROS [201]. Significantly, Logu *et al.* demonstrated that allyl isothiocyanate, a TRPA1 agonist, was found to stimulate the release of ROS from HEK293 cells [156]. This suggests that TRPA1 not only responds to ROS as a downstream target but may also contribute to the generation of ROS or modulation of ROS levels. Following pSNI, NOX2-dependent oxidative bursts in infiltrating macrophages around damaged nerves activate the TRPA1/NOX1/ROS pathway in Schwann cells, subsequently maintaining their recruitment to macrophages and targeting TRPA1 on adjacent nociceptors to improve their efficacy [156]. This regulatory mechanism is also involved in the maintenance of chronic ethanol intake-induced mechanical pain and nitroglycerin-induced trigeminal neuralgia [202, 203]. These data indicate the existence of a cycle that perpetuates an oxidative stress status and involves the mutual induction of ROS generation and TRPA1 expression. Therefore, focusing on controlling or inhibiting these two factors seems to be a highly intriguing therapeutic strategy. Indeed, disruption of the indirect autostimulation circuit by TRPA1 knockout or pharmacological inhibition significantly reduces ROS production in the sciatic nerves of CCI rats, thereby exerting a potent analgesic effect [204].

ATP acts through the ligand-gated ion channel receptor purinergic type 2X (P2X) and metabolism-related G protein-coupled receptor purinergic type 2Y (P2Y) signaling pathways. The activation of these receptors induces the generation of ROS and alters antioxidant defenses, regulating the redox biology of cells [205]. A previous experiment that used P2X7R knockout mice revealed that P2X7R has a partial pronociceptive effect in CFA-induced inflammation and SNI-induced NP [206]. This facilitating role of P2X7R in nociception was further confirmed by a study showing that intrathecal administration of the P2X7R agonist BzATP markedly induces ROS expression and spontaneous nociceptive behavior. More importantly, PBN alleviates BzATP-induced pain behavior without rescuing P2X7R activation. All these results suggest that P2X7R is upstream of ROS dysregulation and is the critical mediator of pathogenic inducement [207]. This is consistent with the *in vitro* findings of Apolloni *et al.*, who showed that P2X7R stimulates the translocation of p67phox and recruitment of Rac1 to induce NOX2-derived ROS production [208]. In cultured neuronal SH-SY5Y cells, pharmacological inhibition or knockdown of P2Y6R prevents increases in ROS and malondialdehyde levels and the downregulation of superoxide dismutase 1 expression following 1-methyl-4-phenylpyridinium stimulation [209]. Based on these data, P2Y6R may be implicated in chronic pain. Unsurprisingly, the P2Y6R antagonist MRS2578 was found to play a key role in attenuating pain behavior in CCI rats due to its ability to inhibit oxidative stress [210]. Moreover, Cirillo *et al.* demonstrated that administration of OxATP reversed SNI-induced glial activation, reduced expression of vesicular GABA transporter and glutamate transporter 1, and increased expression of vesicular glutamate transporter 1, thereby preventing neuropathic behavior [211]. These findings provide valuable insights into the role of ATP in chronic pain.

Toll-like receptor-4 (TLR4) acts as a pathogen-associated molecular pattern receptor. *In vitro* studies conducted by Park *et al.* using a luciferase assay revealed that the Toll-IL-1R domain of TLR4 binds to the COOH-terminal region of NOX4 and regulates its expression [212]. Heme is supposed to be an endogenous agonist of TLR4 [213]. This was confirmed *in vitro* in sickle microglial cells where heme increased endogenous ROS levels. This increase in ROS level was abolished by TAK242 or LPS-RS (a TLR4 inhibitor) [214]. Lei *et al.* showed that in sickle mice, TLR4 transcript levels were significantly increased in the spinal cord, and this alteration was responsible for chronic sickle pain. Specifically, TLR4 knockout or TLR4 inhibitors achieve similar effects in reducing mechanical, heat, and cold sensitivity in sickle mice [214]. Additionally, there is a negative correlation between TLR4 expression and the apoptotic index of neural cells in the prefrontal cortex region exposed to hyperoxia [25]. It should be noted that TLR4 plays an integral role in the body's immune defense. Therefore, periodic pathogen surveillance/clearance is necessary for long-term TLR4 inhibition to prevent unintended consequences related to the duration/extent of infection [215].

Sigma-1 (Sig-1) receptors are widely distributed in the CNS, and their activation enhances intracellular Ca^2+^ signaling by both mobilizing Ca^2+^ release from endoplasmic stores and prompting Ca^2+^ entry through the plasma membrane [216], thus resulting in increased activation of PKC [217], which leads to NOX2 activation by boosting the plasma membrane translocation of p47phox [218]. Indeed, intrathecal injection of PRE084 (a Sig-1R agonist) increases the activation of NOX2 and phosphorylation of NMDA receptor GluN1 subunit (pGluN1) at the Ser896 site in the dorsal horn [219, 220]. Importantly, this practice induces a rapid pronociceptive effect, which can be prevented by pretreatment by intrathecal injection with apocynin (a NOX inhibitor), confirming that NOX is a critical downstream target of Sig-1R-induced nociceptive hypersensitivity in mice. The positive regulation of Sig-1R-induced NOX2 signaling in the spinal cord is also a latent mechanism of injury in NP. Intrathecal injection of BD1047 (a Sig-1R antagonist) inhibits the supposedly boosted spinal NOX2 activation and ROS generation induced by CCI, reducing mechanical pain hypersensitivity. Furthermore, this regulation may partly occur at the posttranslational level, as a single administration of PRE084 to activate Sig-1R did not increase the expression of total p47phox but only the membrane fraction [219]. In addition, the optimal window for the analgesic effects of Sig-1R antagonism is in the induction phase of chronic NP, not the maintenance phase. Based on these findings, preemptive or reactive analgesia to modulate the establishment of chronic pain states and alter the course of NP would be of considerable clinical value in patients who experience direct nerve injury or those with cancer who are treated with chemotherapeutic agents [221].

Interleukin-33 (IL-33) is a nuclear factor derived from the IL1 family that exerts its biological functions by binding to the receptor complex composed of suppression of tumorigenicity 2 (ST2) receptor [222]. As shown in recent studies, it binds to ST2 in peripheral sensory neurons under pain conditions, induces the hyperexcitability of sensory neurons, and mediates nociception [222]. Recent experimental results have confirmed a critical role for IL-33/ST2 in the bidirectional regulation of ROS under pain conditions. ST2 knockout in mice or pharmacological inhibition of ST2 abolishes ROS overexpression and pain hypersensitivity in gout arthritis models, all of which are reversed by exogenous IL-33 [223].

Sphingosine-1-phosphate receptor 2 (S1P2) is a G protein-coupled receptor for sphingosine 1-phosphate (S1P), and its expression in the spinal cord is reduced in mice with CCI-induced NP. This modulation is associated with NP, as intrathecal injection with adeno-associated virus‐S1P2 suppresses ROS generation and reverses established pain [224]. Of note, the analgesic effect of S1P2 appears to be receptor independent, as the hypothesized downregulation of mechanical sensitivity in rats do not occur after SIP1 administration [224]. In addition, the expression of runt-related transcription factor 3, a critical protein involved in the growth and survival of DRG neurons [225], is also regulated in both directions by S1P2, with S1P2 knockdown or overexpression down- or upregulating runt-related transcription factor 3 levels, respectively [224].

Numerous studies have shown that the adverse effects of endothelin signaling in chronic pain are dependent on superoxide anion production, and endothelin receptor antagonists inhibit oxidative stress and reverse KO_2_-induced painful behaviors [226, 227].

MicroRNAs (miRNAs) are involved in a variety of physiological and pathological processes and play important roles in the epigenetic regulation of gene expression, in addition to exerting effects at the transcriptional and posttranscriptional levels [228]. First, YB Im *et al.* confirmed a causal role for NOX4 in NP. Next, they investigated the importance of microRNA23b (miR23b) in NP, as it directly represses the translation of its target gene NOX4 by binding to complementary target mRNAs to elucidate the underlying upstream regulatory mechanism. The level of miR23b in the spinal cords of female mice with SCI-induced NP was significantly lower than that in wild-type animals. After an intrathecal injection of miR23b, the mechanical pain threshold was significantly increased [229]. Moreover, in a rescue experiment, NOX4 knockout occluded the anti-miR23b analgesic effect, confirming the involvement of miR23b mediated NOX4 regulation in NP [229]. Miao *et al.* also proposed that NOX4 may be a miR155 target in the context of pain. In oxaliplatin-induced NP, they quantified the expression of NOX4 and found that it was significantly upregulated in the SDH after oxaliplatin treatment. They analyzed miR155 expression in the same animals and observed a steady increase in miR155 expression, suggesting a causal relationship between miR155 and NOX4. Intrathecal injection of a miR155 inhibitor reduced NOX4 expression in the SDH and significantly alleviated pain behaviors in rats [18]. Similarly, SCI induced a fast and steady increase in miR-155 expression in the early postoperative period, and these phenomena disappeared in the absence of NOX2 [155]. These results suggest that miR-155 may present an integrative role between NOX2 and NOX4 events in ROS-induced NP, and we can regulate the expression of multiple genes through one miRNA or finely regulate the expression of a certain gene through a combination of several miRNAs, thereby affecting a wide range of targets in the pain pathways [230]. Because of the conserved of miRNA properties across animal species and their sequence specificity, as well as the easy delivery of peptide nucleic acids used to suppress miRNAs, miRNAs have inherent advantages for drug targeting [231].

## 4 TARGETING PEROXISOME FOR CHRONIC PAIN THERAPY

Various FAD-dependent oxidoreductases in peroxisomes are the main sources of cellular generation of H_2_O_2_ [232]. Peroxisomes are equipped with enzymatic and nonenzymatic antioxidant systems to maintain their own redox balance [233]. D-amino acid oxidase (DAAO), the earliest enzyme detected in peroxisomes [234], is widely present in pain perception areas of both the brain and spinal cord [235, 236]. It catalyzes the oxidative deamination of d-amino acids in the following manner to generate the catalytic cycle byproduct H_2_O_2_ *in vitro* and *in vivo*: R-CH(NH_2_)-COOH + O_2_ + H_2_O → R – CO_2_ - COOH + NH_3_+ H_2_O_2_ [234]. It plays an important role in chronic pain transmission and transduction due to augmented production of H_2_O_2_, including in formalin-induced tonic pain, bone cancer pain, SNI-induced mechanical allodynia and sleep-deprived mechanical hypersensitivity as well as morphine tolerance. Evidence for this phenomenon includes the fact that the levels of DAAO and ROS are increased in the pain models described above and that genetic ablation or pharmacologic blockade of DAAO attenuates pain behaviors [235, 237-243]. Interestingly, in CCI and trigeminal neuropathic rats, exogenous DAAO exerts an antinociceptive effect [244-246]. A plausible explanation for this finding is that D-serine is an endogenous agonist of NMDA, which is involved in central sensitization, and the D-serine level decreases as a result of the degradation of D-serine by DAAO [247]. Unfortunately, the recent evidence does not fully explain the dual roles of DAAO in the occurrence and development of chronic pain.

## CONCLUDING REMARKS AND FUTURE PERSPECTIVES

Recent studies have challenged the traditional approach of controlling pain through ROS scavenging. Instead, researchers are now focusing on controlling the sources of ROS. While inhibiting NOX has been a primary focus, its control should extend beyond direct inhibition. Novel treatments regulate NOX by preventing abnormal activation *via* the assembly of each subunit or targeting the upstream mechanism involved in the signaling pathway. Additionally, maintaining normal electron transport in the MRC and modulating mitochondrial mass regulation have been shown to be promising therapeutic strategies for chronic pain. This article reviews preclinical evidence from these types of studies to provide a broader perspective on the treatment of chronic pain (Table **1**). However, these findings also raise further questions. First, considering that researchers have mainly focused on reducing ROS levels to relieve pain, the specific mechanisms through which these processes activate the MRC or NOX need to be further examined. Second, due to the complex nature of oxidative stress responses, relying solely on targeted molecular interventions may be too simple of an approach. A deeper understanding of the redox biology involved in chronic pain is essential for effectively managing it through antioxidant therapy. Third, while most studies rely on rodent models, the differences between animal species present significant challenges. To overcome these challenges, transgenic animals that express human receptors can be used to minimize these differences. Fourth, although the generation and regulation of ROS have been discussed individually, it is challenging to determine which specific part of the ROS generation process produce the effect. How ROS in different subcellular compartments communicate and affect cellular functions requires further exploration. Fifth, inhibiting certain molecules with various physiological functions may result in wide-ranging molecular and cellular consequences. Further evaluation is needed to determine whether they can serve as potential therapeutic targets after intervention. Sixth, despite the crucial role of certain pathways or molecules, such as diacylglycerol/PKC, in regulating ROS [248], there is a noticeable lack of specific studies investigating the diacylglycerol/PKC-NADPH oxidase pathway in pain management. It is crucial for future research to extensively explore this pathway, aiming to unravel its potential contributions and underlying mechanisms, which will provide valuable insights into the involvement of ROS in pain processes and open up new avenues for the development of pain management strategies. Encouragingly, the discovery of new pain-relieving effects in existing drugs brings great potential for drug repurposing [249]. By optimizing these drugs as a skeletal structure, the time needed for early-stage compound discovery and optimization can be significantly reduced. Moreover, with clear pharmacokinetic properties and safety parameters, the risk of failure in clinical trials can be effectively minimized.

Although we have discussed the targets involved in regulating ROS for pain management separately, it is undeniable that there are associations among some of these targets. For instance, there exists a complex interplay between ATP, P2X7 receptors, and AMPK, enabling the modulation of multiple signaling pathways with a common target ROS [250]. As such, multi-level regulation including ROS can be achieved by modulating any of the components within ATP, P2X7 receptors, or AMPK.

Overall, ROS have also been reported to exert beneficial effects, and both the differentiation and polarization of CNS neurons depend on a certain level of ROS [184]. Physiological ROS concentrations in specific subcellular sites play important regulatory roles in maintaining cellular homeostasis through protein phosphorylation, transcription factor regulation and signaling functions. Mastering the activation pattern of ROS by monitoring the generation of specific ROS in specific subcellular components and under specific conditions and then administering antioxidant treatment to specific microregions in cells will be beneficial for our management of ROS in the precise treatment of chronic pain. In addition, the specificity of NADPH-derived ROS-mediated signal transduction is related to the localization of NOX isoforms and regulatory subunits in specific subcellular compartments [251]. Whether ROS activated by different signaling pathways have different signatures may be an interesting topic.

## Figures and Tables

**Table 1:** Summary of therapeutic potential of following targets or pathways in chronic pain.

**-**		**Target**		**Drug**		**Model**		**Treatment Strategy**		**Effects**		**Mechanisms**	**References**
Mitochondria-related analgesic targets		Complex I		Rotenone (complex I inhibitor)		VCR, ddC, and STZ-induced neuropathic pain rats		Rotenone (1, 2, 5 and 10 μg, i.d.) was administered from day 5 to 8 after VCR, ddC, and STZ injection.		PWT↑		Restore MRC dysfunction	[56]
						TNFα-induced inflammatory pain rats		Rotenone (1, 2, 5 and 10 μg, i.d.) was co-injected with TNFα.		PWT↑		Restore MRC dysfunction	[56]
						Paclitaxel-induced neuropathic pain rats		Rotenone (3 mg/kg, i.p.) was administered after paclitaxel injection.		PWT↓		MRC dysfunction	[59]
						Oxaliplatin-induced neuropathic pain rats		Rotenone (3 mg/kg, i.p.) was administered after oxaliplatin injection.		PWT↓		MRC dysfunction	[59]
		Complex II		3-Acetylphenyl N-(P-Tolyl) carbamate (complex II inhibitor)		VCR, ddC, and STZ-induced neuropathic pain rats		3-Acetylphenyl N-(P-Tolyl) carbamate (1, 2, 5 and 10 μg, i.d.) was administered from day 5 to 8 after VCR, ddC, and STZ injection.		PWT↑		Restore MRC dysfunction	[56]
						TNFα-induced inflammatory pain rats		3-Acetylphenyl N-(P-Tolyl) carbamate (1, 2, 5 and 10 μg, i.d.) was co-injected with TNFα.		PWT↑		Restore MRC dysfunction	[56]
		Complex III		Antimycin A (complex III inhibitor)		VCR, ddC, and STZ-induced neuropathic pain rats		Antimycin A (1, 2, 5 and 10 μg, i.d.) was administered from day 5 to 8 after VCR, ddC, and STZ injection.		PWT↑		Restore MRC dysfunction	[56]
						Paclitaxel-induced neuropathic pain mice		Antimycin A (0.2 and 0.4 mg/kg, i.p.) was administered once daily for 7 days before and during paclitaxel treatment on days -1 to 5.		PWT↑		Restore MRC dysfunction	[58]
						TNFα-induced inflammatory pain rats		Antimycin A (1, 2, 5 and 10 μg, i.d) was co-injected with TNFα.		PWT↑		Restore MRC dysfunction	[56]
		Complex IV		Sodium cyanide (complex IV inhibitor)		VCR, ddC, and STZ-induced neuropathic pain rats		Sodium cyanide (1, 2, 5 and 10 μg, i.d.) was administered from day 5 to 8 after VCR, ddC, and STZ injection.		PWT↑		Restore MRC dysfunction	[56]
						TNFα-induced inflammatory pain rats		Sodium cyanide (1, 2, 5 and 10 μg, i.d) was co-injected with TNFα.		PWT↑		Restore MRC dysfunction	[56]
		Complex V		Oligomycin (complex V inhibitor)		VCR, ddC, and STZ-induced neuropathic pain rats		Oligomycin (1, 2, 5 and 10 μg, i.d.) was administered from day 5 to 8 after VCR, ddC, and STZ injection.		PWT↑		Restore MRC dysfunction	[56]
						TNFα-induced inflammatory pain rats		Oligomycin (1, 2, 5 and 10 μg, i.d) was co-injected with TNFα.		PWT↑		Restore MRC dysfunction	[56]
						Paclitaxel-induced neuropathic pain rats		Oligomycin (10 mg/kg, i.p.) was administered after oxaliplatin injection.		PWT↓		MRC dysfunction	[59]
						Oxaliplatin-induced neuropathic pain rats		Oligomycin (10 mg/kg, i.p.) was administered after oxaliplatin injection.		PWT↓		MRC dysfunction	[59]
		CoQ		Idebenone (CoQ10 analog)		Carrageenan-induced inflammatory pain rats		Idebenone (1, 5 and 10 mg/kg, i.p.) was administered 15 minutes before the administration of the stimulus.		PWL↑		ROS↓ MnSOD nitration↓	[67]
		CA		5b (CAVA inhibitor)		Paclitaxel-induced neuropathic pain mice		5b (100 mg/kg, p.o.) was administered on day 10 or from day 1 to 15 after paclitaxel injection.		PWL↑		MMP↓ Microglia activation↓ Astrocyte activation↓	[75]
				5d (CAVB inhibitor)		Paclitaxel-induced neuropathic pain mice		5d (100 mg/kg, p.o.) was administered on day 10 or from day 1 to 15 after paclitaxel injection.		PWL↑		MMP↓ Microglia activation↓ Astrocyte activation↓	[75]
		FAM173B		AS-mFAM173B		CFA-induced inflammatory pain mice		AS-mFAM173B was administered from day 5 to 10 after the CFA injection.		PWL↓ PWT↓		MMP↑ ROS↑ Microglia activation↑	[77]
						SNI-induced neuropathic pain mice		AS-mFAM173B was administered from day 1 to 9 after the SNI injury.		PWL↓ PWT↓		MMP↑ ROS↑ Microglia activation↑	[77]
		DRP1		AS-DRP1		Remifentanil-induced hyperalgesia rats		AS-DRP1 (10 μg, i.t.) was administered 30 minutes before remifentanil infusion.		PWT↑ PWL↑		DRP1↓ ROS↓ NMDA↓	[88]
				Mdivi-1 (DRP1 inhibitor)		HIVgp120-induced neuropathic pain rats		Mdivi-1 (3 μl, i.t.) was administered from day 1 to 3 after gp-120 treatment.		PWT↑		DRP1↓ ROS↓	[90]
				2-Bromopalmitate (palmitoylation inhibitor)		CFA-induced inflammatory pain rats		2- Bromopalmitate (0.1 and 0.5 mg/kg, i.t.) was administered one day before the CFA injection.		PWT↑		DRP1↓ OPA1↑ ROS↓ IL-1β↓ TNFα↓ Astrocyte activation↓	[93]
Mitochondria-related analgesic targets		DRP1		Mito -TEMPO		CCI-induced neuropathic pain rats		Mito-TEMPO (0.7 mg/kg, i.p.) was administered from day 7 to 21 after CCI injury.		PWT↑ PWL↑		DRP1↓ FIS1↓ OPA1↑ MFN1↑ ROS↓	[91]
		PGC-1α		Recombinant PGC-1α		Morphine-induced morphine-tolerant rats		Recombinant PGC-1α (20 ng, i.t.) was administered 30 minutes before morphine injection.		PWT↑ PWL↑		PGC-1α↑ ROS↓	[17]
				ZLN005 (PGC-1α activator)		SNI-induced neuropathic pain rats		ZLN005 (50 μg, i.t.) was administered after SNI injury for 7 days.		PWT↑		PGC-1α↑ ROS↓	[100]
		NRF2		RTA-408 (NRF2 activator)		CCI-induced neuropathic pain mice		RTA-408 (1, 5 and 10 μg, i.t.) was administered from day 7 to 11 after CCI injury.		PWT↑ PWL↑		PGC-1α↑ ROS↓	[98]
		MAO		KDS2010 (MAO-B inhibitor)		Paclitaxel-induced neuropathic pain mice		KDS2010 (15, 25 and 50 mg/kg, p.o.) was administered twice daily after paclitaxel injection for 14 days.		PWT↑		ROS↓ Astrocyte activation↓ Restores sIPSCs	[105]
		5-HT(1F)		Lasmiditan (5-HT(1F) receptor agonist)		SNI-induced neuropathic pain rats		Lasmiditan (100 and 200 μg, i.t.) was administered after SNI injury for 12 days.		PWT↑		PGC-1α↑ ROS↓	[100]
		ADRB2		Formoterol fumarate dihydrate (ADRB2 agonist)		Paclitaxel-induced neuropathic pain rats		Formoterol (5, 25 and 50 μg, i.t.) was administered on the 14^th^ day after the first injection of paclitaxel.		PWT↑		PGC-1α↑ NRF1↑ TFAM↑ ROS↓	[99]
		P53		Pifithrin-α (P53 selective inhibitor)		STZ-induced diabetic neuropathic pain mice		Pifithrin-α (1.1 mg/kg, i.p.) was administered three times weekly for 4 weeks starting on the first day of STZ treatment.		PWT↑ PWL↑		P53↓ Parkin↑ ROS↓	[115]
				Pifithrin-µ (P53 selective inhibitor)		Paclitaxel-and cisplatin-induced neuropathic pain rats		Pifithrin-µ (8 mg/kg, i.p.) was administered 1 hour before paclitaxel or cisplatin injection.		PWT↑		P53↓ MMP↑ ROS↓	[116,117]
		Mst1		Mst1 siRNA		CCI-induced neuropathic pain rats		A 120 μl mixture of gel and siRNA was dripped surrounding the sciatic nerve immediately after the ligation.		PWT↑ PWL↑		MMP↑ ROS↓ p62↓ Parkin↓	[120]
		SIRT1		Ro5-4864 (TSPO agonist)		SNI-induced neuropathic pain rats		Ro5-4864 (2 μg, i.t.) was administered from day 3 to 5 after surgery.		PWT↑		SIRT1↑ PGC-1α↑ Beclin1↓ LC3↓ P62↓	[125]
		SIRT3		LV-SIRT3		STZ-induced diabetic neuropathic pain rats		LV-SIRT3 (10 μl/day, i.t.) was administered for 5 days after 15 days of STZ injection.		PWT↑ PWL↑		FOXOa3↑ MnSOD↑ Catalase↑	[129]
						SNI-induced neuropathic pain rats		LV-SIRT3 (5 μl/day, i.t.) was administered for 5 days after 15 days of STZ injection.		PWT↑ PWL↑		Ac-CypD↓ mPTP↓ MMP↑ ROS↓	[19]
		AMPK		AICAR (AMPK activator)		Cancer-induced bone pain rats		AICAR (1 mg/kg, i.t.) was administered after the CIBP animal model was established.		PWT↑		AMPK↑ ROS↓ DRP1↓ IL-1β↓ NLRP3↓	[141]
				Metformin (AMPK activator)		Non-obese type 2 diabetic mice		Metformin (150 mg/kg, i.p.) was administered after SNI injury for 13 weeks.		PWT↑ PWL↑		AMPK↑ NOX↓ Beclin-1↓ LC3↓ ROS↓	[143]
NOX-related analgesic targets		Rac1		NSC23766 (Rac1 inhibitor)		Burn injury-induced neuropathic pain mice		NSC23766 (5 μg, i.t.) was administered twice daily for 3 days after burn injury.		PWT↑ PWL↑		Rac1↓ Dendritic spine density↓	[176]
						Paclitaxel-induced neuropathic pain rats		NSC23766 (10 μg, i.t.) was administered on days 15, 20 and 25 after paclitaxel treatment.		PWT↑		Rac1↓ Dendritic spines maturity↓	[177]
		APE1/Ref-1		E3330 (selective inhibitor of APE1-redox activity)		CFA-induced inflammatory pain rats		E3330 (10 μl, i.t.) was administered 5 minutes before CFA injection.		PWT↑		APE1↑ Rac1↓ ROS↓ NF-κB↓ IL-6↓	[180]
		RyR		Dantrolene (antagonist of RyR)		HIVgp120-induced neuropathic pain rats		Dantrolene (1, 3 and 10 µm, i.t.) was administered 2 weeks after HIV gp120 injection.		PWT↑		ROS↓	[186]
		Cav-1		Daidzein (Cav-1 specific inhibitor)		STZ-induced diabetic neuropathic pain mice		Daidzein (0.4 mg/kg/day, i.p.) was administered for 2 weeks after 14 days of STZ injection.		PWT↑ PWL↑		Cav-1↑ Rac1↓ NOX2↓ ROS↓ NR2B↓	[189]
		p47phox		Curcumin		STZ-induced diabetic neuropathic pain mice		Curcumin (200 mg/kg, *i.e*.) was administered for 14 days after STZ injection.		PWT↑		p47phox↓ gp91phox↓ ROS↓ MDA↓ SOD↑	[191]
		Hv1		YHV98-4 (Hv1 specific inhibitor)		CFA-induced inflammatory pain mice		YHV98-4 (10 mg/kg, i.p.) was administrated twice daily for 2 days after CFA injection.		PWT↑ PWL↑		Hv1 ↓ ROS↓ SHP-1↑	[199]
						Opioid-induced tolerance and hyperalgesia in mice		YHV98-4 (10 mg/kg, i.p.) was administrated for 6 days after the first injection of CFA.		PWT↑ PWL↑		Hv1↓ Restore ROS-SHP-1-PI3K/ pAKT-CXCL1 pathway	[199]
NOX-related analgesic targets		TRPA1		HC030031 or A967079 (TRPA1 selective antagonist)		pSNL-induced neuropathic pain mice		HC030031 (100 mg/kg, i.p.) or A-967079 (100 mg/kg, i.p.) was administered on day 10 after surgery.		PWT↑ PWL→		TRPA1↓ NOX1↓ ROS↓	[156]
						Glyceryl trinitrin-induced migraine in mice		HC030031 (100 mg/kg, i.p.) or A-967079 (100 mg/kg, i.p.) was administered 0.5 hour before and 1, 3, 4 and 5 hours after glyceryl trinitrin injection.		PT↑		TRPA1↓ NOX1↓ ROS↓	[203]
				A967079 (TRPA1 selective antagonist)		Ethanol-induced neuropathic pain mice		A967079 (100 mg/kg, i.p.) was administered on day 28 after ethanol.		PWT↑		TRPA1↓ NOX1↓ ROS↓	[202]
		P2		OxATP (nonselective P2XR antagonist)		SNI-induced neuropathic pain mice		OxATP (6 mg/kg, i.p.) was administered from day 1 to 21 after surgery.		PWT↑ PWL↑		Microglial activation↓ Astrocytic activation↓ GLT1↓ vGAT↓ vGLUT1↑ P2X7R↓ ROS↓	[211]
				A438079 (P2X7R antagonist)		BzATP-induced nociceptive pain mice		A438079 (100 mg/kg, i.p.) was administered 30 minutes before the injection of BzATP.		PLR↑		P2X7R↓ ROS↓	[207]
				MRS2578 (P2Y6R antagonist)		CCI-induced neuropathic pain rats		MRS2578 (10 μl, i.t.) was administered on days 3, 7 and 14 after surgery.		PWT↑ PWL↑		P2Y6R↓ ROS↓ SOD↑ GSH↑ HO-1↑	[210]
		TLR4		TAK242 (TLR4 inhibitor)		Heme-induced chronic pain in sickle mice		TAK242 (1 mg/kg/day, i.v.) was administered for 5 days after model establishment.		PWT↑ PWL↑		TLR4↓ ROS↓ IL-6↓ Microglial activation↓	[214]
		Sig-1		BD1047 (Sig-1R antagonist)		CCI-induced neuropathic pain rats		BD1047 (100 nmol, i.t.) was administered twice daily on days 0–5 or 15–20 after surgery.		PWT↑ PWL→		Sig-1↓ NOX2↓ ROS↓	[219]
		S1P		S1P2 shRNA		CCI-induced neuropathic pain rats		S1P2 shRNA (2×10^11^ vector genomes per, i.t.) was administered 3 days before CCI surgery.		PWT↓ PWL↓		S1P2↓ ROS↑ IL-1β↓ IL-6↓ CCL2↓ RUNX3↓	[224]
		IL-33/ST2		IL-33 neutralizing antibody		MSU-induced gouty arthritis in mice		IL-33 neutralizing antibody (5 μg, i.p.) was administered 1 hour before and 8, 23 hours after model establishment.		PWT↑		IL-33↓ ROS↓ MDA↓ 4-HNE↓ CXCL1↓ CCL3↓ IL-1β↓ IL-6↓	[223]
				ST2 neutralizing antibody		MSU-induced gouty arthritis in mice		ST2 neutralizing antibody (50 μg, i.p.) and IL-33 neutralizing antibody (300 ng, i.p.) were co-administered 24 hours after model establishment.		PWT↑		ST2↓ ROS↓ MDA↓ 4-HNE↓ CXCL1↓ CCL3↓ IL-1β↓ IL-6↓	[223]
		Endothelin A		Clazosentan (endothelin A receptor antagonist)		KO_2_-induced inflammatory pain mice		Clazosentan (3, 10 and 30 nmol, i.p.) was administered 30 minutes before KO_2_ injection.		PWT↑ PWL↑		ROS↓ IL-1β↓ TNFα↓ MPO↓	[227]
		Endothelin B		BQ-788 (endothelin B receptor antagonist)		KO_2_-induced inflammatory pain mice		BQ-788 (3, 10 and 30 nmol, i.p.) was administered 30 minutes before KO_2_ injection.		PWT↑ PWL↑		ROS↓ MPO↓	[227]
		miR23b		-		SCI-induced neuropathic pain rats		miR23b (10 μM, i.t.) was administered after surgery.		PWT↑ PWL↑		NOX4↓ ROS↓ COX2↓ TNFα↓ IL-1β↓ caspase-3↓ Bcl-2↑ GFAP↓ Iba1↓	[229]
		miR155		5′AAU UAC GAU UAG CAC UAU CCC CA-3′ (miR155 inhibitor)		Oxaliplatin-induced neuropathic pain rats		5′AAU UAC GAU UAG CAC UAU CCC CA-3′ (2 μg, i.t.) was administered for 5 days after oxaliplatin injection.		PWT↑ PWL↑		NOX4↓ NRF2↑ ROS↓ NQO1↑	[18]
Peroxisome-related analgesic targets		DAAO		CBIO, sodium benzoate, AS-057278 (DAAO selective inhibitors)		REMSD-induced pain rats		CBIO (3 μg, i.t.), sodium benzoate (30 and 100 μg, i.t.) and AS-057278 (3 and 10 μg, i.t.) were administered 48 hours after REMSD, respectively.		PWT↑		DAAO↓ ROS↓	[240]
						Morphine-induced analgesic tolerance rats and mice		CBIO (10 mg/kg, s.c.), AS057278 (40 mg/kg, s.c.) and sodium benzoate (400 mg/kg, s.c.) were administered for 7 days after the first injection of morphine, respectively.		PLR↑ TFL↑ PWL↑		DAAO↓ ROS↓	[241]
								CBIO (1, 3, and 10 mg/kg, s.c.), AS057278 (3, 10 and 30 mg/kg, s.c.) and sodium benzoate (10, 30, 100 and 300 mg/kg, s.c.) were administrated 20 minutes before morphine challenge.		PLR↑ TFL↑ PWL↑		DAAO↓ ROS↓	[241]
Peroxisome-related analgesic targets		DAAO		Sodium Benzoate (DAAO selective inhibitor)		Acetic acid-induced visceral pain mice		Sodium benzoate (400 mg/kg, i.v.) was administered 30 minutes before the administration of the stimulus.		TFL↑ PLR↑		DAAO↓	[242]
						Formalin-induced inflammatory pain mice		Sodium benzoate (400 mg/kg, i.v.) was administered 30 minutes before the administration of the stimulus.		TFL↑ PLR↑		DAAO↓	[242]
				SUN (DAAO inhibitor)		SNI-induced neuropathic pain rats		SUN (3, 10 and 30 mg/kg, p.o.) was administered from day 11 to 14 after surgery.		PWL↑		DAAO↓	[243]
						CCI-induced neuropathic pain rats		SUN (3, 10 and 30 mg/kg, p.o.) was administered from day 13 to 17 after surgery.		PWL↑		DAAO↓	[243]
						CFA-induced inflammatory pain rats		SUN (3, 10 and 30 mg/kg, p.o.) was administered after CFA injection.		PWL↑		DAAO↓	[243]

**Abbreviations:** VCR: vincristine; ddC: 2’,3’-dideoxycytidine; STZ: streptozotocin; MRC: mitochondrial respiratory chain; CoQ: coenzyme Q; PWL: paw withdrawal latency; MnSOD: manganese superoxide dismutase; CA: carbonic anhydrase; AS-mFAM173B: antisense oligodeoxynucleotides against mFAM173B; CFA: complete Freund's adjuvant; PWT: paw withdrawal threshold; MMP: mitochondrial membrane potential; SNI: spared nerve injury; MONPs: manganese oxide nanoparticles; TFL: tail flick latency; COX2: cyclooxygenase 2; DRP1: dynamin-related protein 1; AS-DRP1: antisense oligodeoxynucleotides against DRP1; NMDA: N-methyl-D-aspartate; OPA1: optic atrophy 1; IL-1β: interleukin-1β; TNFα: tumor necrosis factor α; CCI: chronic constriction injury; FIS1: mitochondrial adaptor fission 1; MFN1: Mitofusins1; PGC-1α: peroxisome proliferator-activated receptor-gamma coactivator-1alpha; MAO: monoamine oxidase; 5-HT: 5-hydroxytryptamine; ADRB2: β2-adrenoreceptor; NRF1: nuclear respiratory factor 1; TFAM: mitochondrial transcription factor A; AMPK: adenosine monophosphate-activated protein kinase; CIBP: cancer-induced bone pain; NLRP3: NOD-like receptor thermal protein domain associated protein 3; FOXO: forkhead homeobox type O; NOX: nicotinamide adenine dinucleotide phosphate oxidase; Mst1: mammalian ste20-like kinase1; siRNA: small interfering RNA; SIRT1: sirtuin-1; TSPO: translocator protein; SNL: spinal nerve ligation; LC3: microtubule-associated protein 1 light chain 3; SIRT3: sirtuin-3; FOXOa3: forkhead homeobox type O 3a; Ac-CypD: cyclophilin D; mPTP: mitochondrial permeability transition pore; APE1: apurinic/apyrimidinic endonuclease 1; Ref-1: redox factor-1; NF-κB: nuclear factor kappa B; IL-6: interleukin 6; RyR: ryanodine receptor; Cav-1: caveolin-1; NR2B: NMDA receptor 2B; KO_2_: superoxide anion donor; NRF2: nuclear factor E2-related factor 2; HO-1: heme oxygenase-1; MDA: malondialdehyde; SOD: superoxide dismutase; LPS: lipopolysaccharide; TPPU: trifluoromethoxyphenyl-3-(1-propionylpiperidin-4-yl) urea; SEH: soluble epoxide hydrolase; ASC: apoptosis-associated speck-like protein containing a caspase activation and recruitment domain; NLRC4: nucleotide-binding and oligomerization domain-like receptor C4; iNOS: inducible nitric oxide synthase; nNOS:neuronal nitric oxide synthase; CCL2: C-C motif chemokine ligand 2; IL-33: interleukin-33; ST2: suppression of tumorigenicity 2 receptor; MSU: monosodium urate; 4-HNE: 4-hydroxynonenal; CXCL1: C-X-C motif chemokine ligand 1; CCL3: C-C motif chemokine ligand 3; S1P: sphingosine 1-phosphate; S1P2: sphingosine‐1‐phosphate receptor 2; RUNX3: runt‐related transcription factors 3; S1P1: sphingosine-1-phosphate receptor 1; Hv1: hydrogen voltage-gated channel 1; SHP-1: Src homology 2 domain-containing tyrosine phosphatase 1; PI3K: phosphatidylinositol-3 kinase; pAKT: phosphorylated protein kinase B; TRPA1: transient receptor potential ankyrin 1; P2: purinergic type 2; pSNL: partial sciatic nerve ligation; PT: periorbital threshold ; MPO: myeloperoxidase; sIPSCs: spontaneous inhibitory postsynaptic currents; miR23b: microRNA23b; SCI: spinal cord injury; GFAP: glia fibrillary acidic protein; miR155: microRNA155; NQO1: nicotinamide adenine dinucleotide phosphate quinone oxidoreductase-1; GLT1: glutamate transporter 1; vGAT: vesicular gamma-aminobutyric transporter; vGLUT1: vesicular glutamate transporter 1; P2X7R: P2X7 receptor; PLR: paw licking response; P2Y6R: P2Y6 receptor; GSH: glutathione; TLR4: toll-like receptor 4; Sig-1: sigma-1; Sig-1R: sigma-1 receptor; DAAO: d-amino acid oxidase; TFL: tail flick latency; REMSD: rapid eye movement sleep deprivation; SUN: 4H-furopyrrole-5-carboxylic acid; CBIO: 6-chlorobenzo[d]isoxazol-3-ol; i.d.: intradermally; i.g.: intragastrically; i.p.: intraperitoneally; i.t.: intrathecally; s.c.: subcutaneous; i.v.: intravenous; p.o.: oral; ↓: downregulated; ↑: upregulated; →: no effect.

## LIST OF ABBREVIATIONS

5-HT: 5-hydroxytryptamine
AMPK: Adenosine Monophosphate-activated Protein Kinase
APE1: Apurinic/Apyrimidinic Endonuclease 1
CA: Carbonic Anhydrase
Cav-1: Caveolin-1
CCI: Chronic Constriction Injury
CCL2: C-C motif Chemokine Ligand 2
CFA: Complete Freund's Adjuvant
CNS: Central Nervous System
CoQ: Coenzyme Q
CREB: cAMP-response Element-binding Protein
DAAO: d-Amino Acid Oxidase
DRG: Dorsal Root Ganglia
DRP1: Dynamic-associated Protein 1
ER: Endoplasmic Reticulum
FAD: Flavin Adenine Dinucleotide
FADH_2_: 1,5-Dihydroflavin Adenine Dinucleotide
FIS1: Mitochondrial Adaptor Fission 1
GABA: Gamma-aminobutyric
Hv1: Hydrogen Voltage-gated Channel 1
IL-33: Interleukin-33
MAO: Monoamine Oxidase
mEPSCs: Miniature Excitatory Postsynaptic Currents
miR155: MicroRNA155
miR23b: MicroRNA23b
miRNAs: MicroRNAs
MMP: Mitochondrial Membrane Potential
MRC: Mitochondrial Respiratory Chain
Mst1: Mammalian ste20-like kinase1
NADPH: Nicotinamide Adenine Dinucleotide Phosphate
NF-κB: Nuclear Factor-kappa B
NLRP3: NOD-like Receptor Thermal Protein Domain Associated Protein 3
NMDA: N-methyl-D-aspartate
NP: Neuropathic Pain
NRF1: Nuclear Respiratory Factor 1
NRF2: Nuclear Factor Erythroid 2-related Factor 2
OPA1: Optic Atrophy 1 Protein
P2X: Purinergic Type 2X
P2Y: Purinergic Type 2Y
PGC-1α: Peroxisome Proliferator-activated Receptor-gamma Coactivator-1alpha
PKC: Protein Kinase C
pSNL: Partial Sciatic Nerve Ligation
Ref-1: Redox Factor-1
RNS: Reactive Nitrogen Species
ROS: Reactive Oxygen Species
RyR: Ryanodine Receptor
S1P: Sphingosine 1-phosphate
S1P2: Sphingosine-1-phosphate Receptor 2
SCI: Spinal Cord Injury
SDH: Spinal Dorsal Horn
sEPSCs: Spontaneous Excitatory Postsynaptic Currents
Sig-1: Sigma-1
sIPSCs: Spontaneous Inhibitory Postsynaptic Currents
siRNA: Small Interfering RNA
SIRT: Sirtuins
SNI: Spared Nerve Injury
SNL: Sciatic Nerve Ligation
ST2: Suppression of Tumorigenicity 2 Receptor
STZ: Streptozotocin
TFAM: Mitochondrial Transcription Factor A
TLR4: Toll-like-receptor-4
TNFα: Tumor Necrosis Factor α
TRPA1: Transient Receptor Potential Ankyrin 1
UCP: Uncoupling Protein
